# Environmental and capacity drivers of health function: a full perspective of the healthy aging framework

**DOI:** 10.3389/fpubh.2025.1659061

**Published:** 2025-09-24

**Authors:** Jingjing Cai, Qunlong Wang, Minmin Jiang, Hongying Zhu, Lu Li

**Affiliations:** ^1^School of Public Affairs, Zhejiang Shuren University, Hangzhou, China; ^2^Zhejiang Modern Service Industry Research Centre, Hangzhou, China; ^3^Shulan International Medical College, Zhejiang Shuren University, Hangzhou, China; ^4^The Institute of Social and Family Medicine, School of Medicine, Zhejiang University, Hangzhou, China; ^5^Zhejiang Urban Governance Research Centre, Hangzhou, China

**Keywords:** surrounding characteristics, ability characteristics, desired states, age-friendly environment, social environment

## Abstract

**Objectives:**

This paper explores how health capacity and environmental factors work together on health function among Chinese older adults.

**Methods:**

A total of 2,788 Chinese older adults was randomly selected, with a valid response rate of 96.4% (*n* = 2,688). Individual, family and social environments were assessed as mediators or moderators in the relationship between health capacity and health function. Structural equation models (SEM) were used to examine the pathways of determinants to health function.

**Results:**

We found that an individual’s capacities and environmental factors had associations with health function in aging settings. Specifically, older adults who reported higher scores in health capacities, age-friendly family and social environment, and higher level of individual life expectation were more likely to experience higher level of health fucntion. Regarding the pathway to health function, age-friendly family (APGAR) and social environments acted as moderators in the relationship between health capacity and health function. The indirect way of health capacity (path coef. =0.269) is stronger than the direct way (path coef. =0.079). The role of social environment (path coef. =0.409) is the predominant in these pathways.

**Discussion:**

This study suggests that both capacity and environmental factors are vital to maintain older adults’ health function. And the construction of an age-friendly environment, especially social environment, contributes a lot to healthy aging.

## Introduction

The World Health Organization (WHO) proposed Healthy Aging as a response to the rapid aging trend and the challenge it poses in the way of social care. Healthy Aging highlights the process of developing and maintaining the functional ability of older adults to ensure well-being in later life (1). The concept of healthy aging takes functional ability as the key factor to promote well-being. It represents the things or states that older adults could do or achieve, which is also referred to as health function, reflecting the valued beings/states that matter for older adults. According to the theory of demand, when a lack hinders the realization of one’s valued beings/states, it would raise one’s desire to make up for this lack (2, 3). It is the function that reflects one’s real demand. Actually, before healthy aging, health outcomes (e.g., quality of life, and morbidity) was often chosen as the individual-development goal or an end-point event in the health promotion studies (4, 5). Health outcomes is a static picture resulting from the dynamic process of health function (6). Neither the well-being-oriented or diseases-oriented health outcomes assessment provides the direct intervention targets for older adults. But in the healthy aging framework, it uses health function to respond to “What life do older adults want?,” and sets a function-oriented pathway involving capacity and environment to answer “How to make older adults’ life?.” According to healthy aging framework, health capacity is defined as “the composite of an individual’s physical and mental attributes,” and health function was defined as “the realization of capacity in achieving specific, valued life outcomes.”

From theoretical perspective, healthy aging framework tells a full story of how to ensure the well-being in later life. However, it needs much more effort to translate the theoretical ideas to empirical applications. The first is to determine the drivers of health function from a framework perspective, and the second is to explore the pathway to health function. So the main question of this study is “what capacity and environment could promote health function, and how?” Up to now, the explorations of healthy aging framework is mainly about the identification of capacity and environment factors that related to health function, and pooled a wide range of factors. For example, a national population survey in Ecuador found some individual inherent capacity characteristics (e.g., gender, educational attainment, and economic status) were closely related to healthy aging levels (7). And a meta-analysis found that physical activity, diet, self-awareness, outlook/attitude, life-long learning, faith, social support, financial security, community engagement, and independence were key determinants related to healthy aging (8). Also, WHO launched the Integrated Care for Older People (ICOPE) in 2019, which comprehensively considered clinical evidence and proposed 5 capacities closely related to function, namely cognition, locomotion, vitality, psychology, and sensory capacity (9, 10). And some researchers put efforts to confirm the role of capacity in healthy aging (11–15). Nonetheless, the key assumption in the healthy aging framework, that health capacity and environment work together to health function, is underexplored.

Does this assumption need to be studied? Back to the concept of healthy aging, health function is determined by health capacity, environment, and the interaction between health capacity and environment. Previous studies argued that environmental conditions may facilitate or hinder one’s ability in activities or participation (16, 17). Supportive neighborhood environments were found to be enablers for daily movements (18). And from older adults’ view, social and physical environment were assumed to be more important than individual behavior (19). It can be inferred that the environment may act as a conversion factor in the relationship between health capacity and health function. As the Capacity Approach Theory suggests, only in the conversion of individual, family, and social surroundings, can one’s ability be fully realized and come into some specific function (20). The existence of such working together mechanism could make healthy aging more possible to realize. Thus, it is reasonable to explore how health capacity work through the environment and then on the health function. Technically, the environment may serve as a moderator or a mediator in the relationship between health capacity and health function.

The aim of this study is to (1) explore the drivers of health function from a full perspective of health capacity and environment, and (2) examine the interaction of environment and health capacity in the healthy aging framework. The conceptual framework of this study is grounded by the healthy ageing framework proposed by WHO (46).

**Figure 1:**
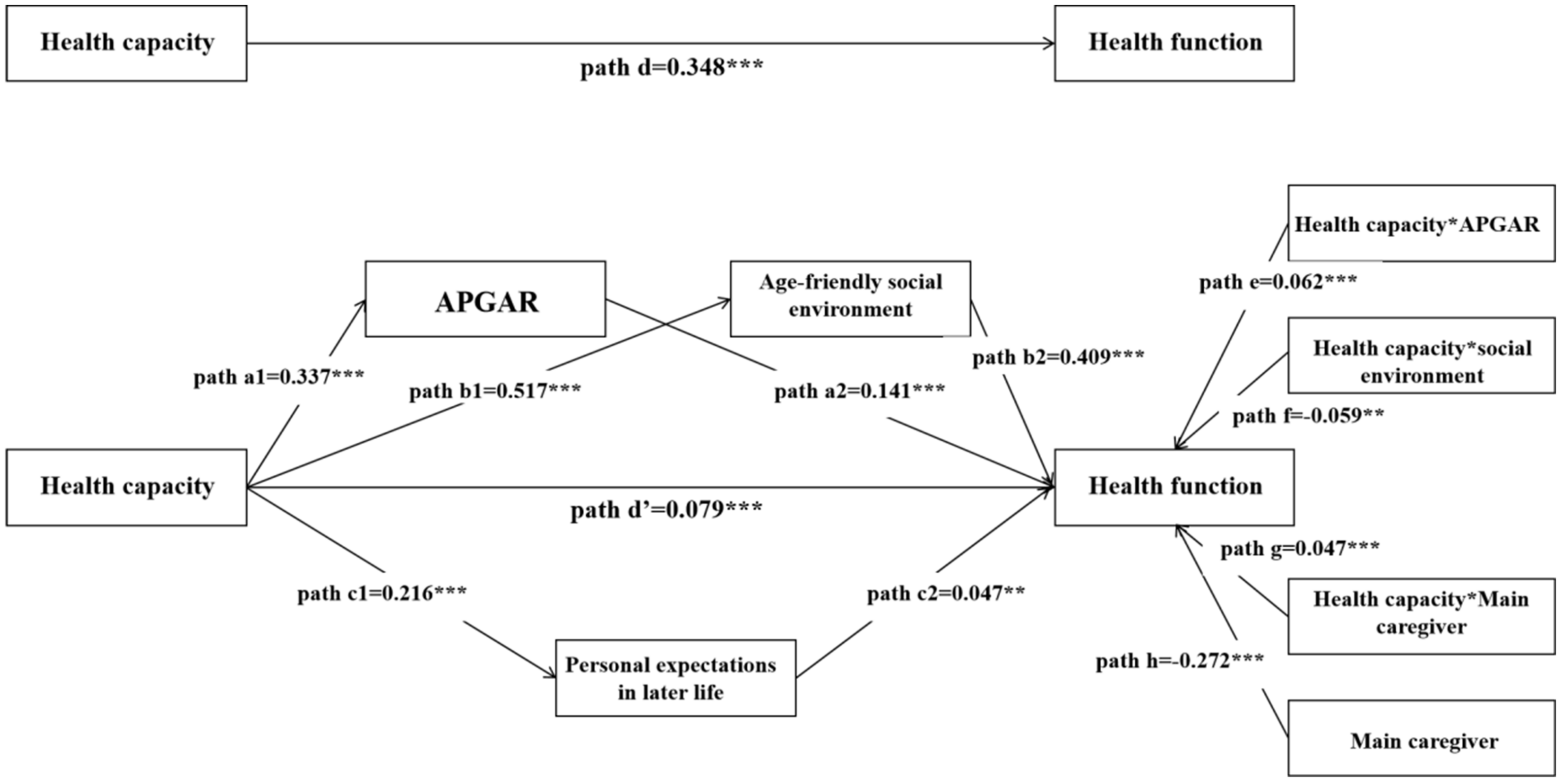
The conceptual framework of this study.

## Materials and methods

### Study design and participants

We conducted a cross-sectional study in the community/village setting in Zhejiang Province from June to September 2022. Zhejiang, a representative province in eastern China during the aging process, had 10.48 million older adults (≥65 years old, 15.71%) at the end of 2024. Participants were recruited using a multi-stage stratified random sampling design. Considering the economic level and the density of older adults, two cities (Hangzhou and Quzhou) were randomly chosen as the study sites in the first step. One district (Gongshu)/county (Kaihua) was selected randomly from each city and in each district/county, two streets/towns were selected randomly. Finally, according to the community/village roster of the Street/Town Civil Affairs/Health Department, multiple communities or villages were randomly selected in each street/town. In these communities/villages, participants were selected randomly from the older adults register. We first invited those selected older adults over the telephone/through community workers’ visits. If anyone refused to participate in, we would do the random selection from older adults register again, until fitting the minimum sample size requirement. According to Sun and Xu (21), $n=\frac{{Z_{\alpha /2}^{2}}^{*}\pi^{*}(1-\pi )}{\delta^{2}}$ , while *α* = 0.05, Zα/2 = 1.96; = 0.1*π*, π = 0.48 (the average health need rate among older adults is 0.48 in Zhejiang Province), the desired minimum sample size was 434 in each site. Also, to fit the overall demographic characteristics of Chinese older adults, the proportion of females and younger older adults in the sample was required to be higher than males and older people. The inclusion criteria included Chinese residents who (1) were aged 60 years or above, (2) could speak Mandarin, and (3) were able to give consent and agreed to participate in the study. Those who did not understand the content of the questionnaire were excluded. Measures to ensure the quality of data please see Part A in the Appendix.

### Data collection and instruments

The questionnaire consisted of 3 sections, including health function and capacity, healthy aging-related environmental factors, and demographic characteristics. To test the applicability of the questionnaire, the cognitive interview was implemented with 30 older adults. This interview required to record whether older adults get the questions correctly and easily, and whether their answers match the preset options. There was no change needed after analyzing the results from the interview. Finally, a face-to-face questionnaire interview was adopted to collect information on older adults who were eligible and willing to participate in.

### Healthy function and capacity

Healthy function and capacity were measured by the Comprehensive Assessment Scale for Health and aging Care Needs (22). This 25-item scale evaluates: (1) Health function (15 items, Likert 5-point): physical, mental, psycho-emotional health, and life perception. The total score ranged from 15 to 75, and higher scores represented better function; (2) Health capacity (10 items): Cognitive ability (0–11), care-seeking ability (0–21), and health choice ability (4–20; Likert scale). Total capacity scores ranged from 4 to 52, the higher the scores, the greater the capacity. The scale demonstrated excellent reliability (Cronbach’s *α* = 0.88; composite reliability = 0.96) and validity (CFI = 0.97, RMSEA = 0.04). This scale was developed according to the healthy aging framework and validated in Chinese settings across urban and rural areas. As a new measurement tool, we did the cross-scale validations and confirmed its criterion validity (see Part A in the Appendix).

### Healthy aging-related environmental factors

Healthy aging-related environmental factors included personal expectation in later life and an age-friendly environment.

The Personal expectation in later life is measured by one question “What state of life do you expect to achieve in the future?.” Drawing on Maslow’s hierarchy of needs theory, the five aspects of expected state of aged care could be dichotomized into basic expectations and developmental expectations.

The age-friendly environment was composed of a family environment and a social environment.

The age-friendly family environment was evaluated via the Family APGAR Index (23, 24) from the family function aspect, and three binary questions from family assistance aspect. The APGAR is a 5-itemscale, measures adaptation, partnership, growth, affection, and resolution of family function. The total score ranged from 0 to 10, with higher scores representing the stronger family function (Cronbach’s *α* = 0.93). Family assistance was measured by asking older adults whether they have received daily affairs support, economic support, or emotional support from family members. Each item was scored on a dichotomous “Yes” or “No” response.

The age-friendly social environment adapted the Age-Friendly Communities Framework recommended by WHO (25), which includes 15 items and 4 domains: accessibility guarantee environment, information dissemination environment, social participant environment, and life security environment. The total score ranges from 0 to 15, with higher scores indicating greater age-friendliness.

### Demographic characteristics

Demographic characteristics information includes three aspects: basic individual characteristics (e.g., age, gender), aged care status (e.g., main caregiver), and health risk characteristics (e.g., chronic disease, medical visit).

### Statistics analysis

The data were summarized by descriptive analyses such as frequency distribution and percentage [*n*(%)] for categorical variables, and Median and inter-quartile range [M(IQR)] for continuous variables. The statistics analysis was composed of two parts: (1) explorations of the determinants of health function, from the perspectives of health capacity and environment factors; and (2) construction of the pathways from health capacity to health function. The first part involved the descriptive analysis, correlation analysis, and the second part involved the pathway analysis.

In the first part, the Mann–Whitney U test and the Chi-square test were adopted to examine the differences in health function, health capacity, and environmental factors by demographic characteristics. The correlations between health function, health capacity, and healthy aging-related environmental factors were examined by using the Mann–Whitney U test and Spearman correlation test. We employed multiple linear regressions to explore the associations between health capacity, environmental factors, and health function. The moderating and mediating analyses were used to examine the role of the environment in the relationship between health capacity and health function. For the robustness of the results, demographic characteristics that were meaningful in univariate analysis were included in the moderation test. Also, for the moderation analysis, it is necessary to first establish the product term of the capacity variable and the moderating variable, and then centralize the continuous variable and the product term before incorporating them into the regression equation. In the second part, structural equation models (SEM) were adopted to further examine the approach to healthy aging (“health capacity-environment-health function”). SPSS version 24 and AMOS 22.0 were used in statistical procedures. A significant level of 5% was adopted.

## Results

### Characteristics of health function and health capacity

Among 2,901 invited, a total of 2,788 older adults agreed to participate in this study, resulting in a response rate of 96.1%. The valid response rate was 96.4% (*n* = 2,688), considering the full response in the key variables.

Supplementary Table 2 shows the descriptive results of health function and health capacity (See Part B in the Appendix). Besides ethics, the scores of health function display significant differences among all the demographic characteristics. A similar distribution in demographic characteristics is found in health capacity. But except for ethics, gender and doctor visits in the past 2 weeks do not have significant associations with health capacity. And Supplementary Table 3 presents the bivariate correlations between health capacity, environmental factors, and health function (see Part B in the Appendix). After descriptive analysis of key variables, muti-variable linear and logistic regressions were used to examine the mediating and moderating role of environmental factors in the relationships between health capacity and health function.

### Mediator examinations of environmental factors in the relationship between health capacity and health function

Table 1 demonstrates the relationships between health capacity, environmental factors, and health function, and examines the mediating role of environmental factors. Table 1 is composed of 6 mediator examinations for each environmental factor. Every mediator examination includes 2 models, and the first model displays the relationship between health capacity and specific environmental factors while the second model shows the relationship between health capacity, specific environmental factors, and health function. The mediator effect is confirmed when these relationships are all significant. After controlling for demographic characteristics, health capacity is positively associated with health function (*B* = 0.44, S. E. = 0.04, *p*<0.001). In the mediator examinations, personal expectations of later life (*B* = 4.08, S. E. = 0.79, *p*<0.001), APGAR (*B* = 0.70, S. E. = 0.07, *p*<0.001), and age-friendly social environment (*B* = 1.49, S. E. = 0.10, *p*<0.001) are positively associated with health function, and all of these factors partly mediated the relationship between health capacity and health function.

**Table 1:** The mediator examinations of environmental factors in the relationship between heath capacity and health function.

Mediator examination 1	Model 1		Model 2	
	Personal expectations in later life		Health function	
Independent variables	OR	95%CI	B	S. E.
Health capacity	1.16***	(1.11, 1.20)	0.410***	0.038
Personal expectations of later life (ref: basic level)			4.084***	0.785
Mediator examination 2	Model 3		Model 4	
	APGAR		Health function	
Independent variables	B	S. E.	B	S. E.
Health capacity	0.131***	0.011	0.331***	0.038
APGAR			0.697***	0.071
Mediator examination 3	Model 5		Model 6	
	Daily affairs support from family members		Health function	
Independent variables	OR	95%CI	B	S. E.
Health capacity	1.12***	(1.09, 1.15)	0.456***	0.038
Daily affairs support from family members (ref: No)			−0.807	0.43
Mediator examination 4	Model 7		Model 8	
	Economic support from family members		Health function	
Independent variables	OR	95%CI	B	S. E.
Health capacity	1.07***	(1.05, 1.10)	0.450***	0.038
Economic support from family members (ref: No)			−0.61	0.426
Mediator examination 5	Model 9		Model 10	
	Emotional support from family members		Health function	
Independent variables	OR	95%CI	B	S. E.
Health capacity	1.07***	(1.05, 1.09)	0.448***	0.038
Emotional support from family members (ref: No)			−0.26	0.351
Mediator examination 6	Model 11		Model 12	
	Age-friendly social environment		Health function	
Independent variables	B	S. E.	B	S. E.
Health capacity	0.101***	0.008	0.292***	0.038
Age-friendly social environment			1.494***	0.098

Each model was controlled after co-variates that significant in the single factor analyses. Durbin-Weston test, residual plot, and P–P plot were used to determine whether the residuals of the multiple linear regression model are independent, and whether they conform to normality and homogeneity of variance. All of the models fit these requirements. Due to space limitations, it will not be displayed here.

### Moderator examinations of environmental factors and demographic characteristics in the relationship between health capacity and health function

Table 2 illustrates the moderator examination results of environmental factors and Supplementary Table 7 (see Part D in Appendix) shows the results of demographic characteristics in the relationship between health capacity and health function. After adding the interaction terms of health capacity and potential moderators, the change of *F* value in each model is significant (*p*<0.001). Table 2 shows, after controlling for all covariates, there is a significant moderating effect of APGAR (*B* = 0.73, S. E. = 0.20, *p*<0.001) and age-friendly social environment (*B* = −0.85, S. E. = 0.18, *p*<0.001) on the relationship between health capacity and health function. Supplementary Table 7 shows, that there is a significant moderating effect of the main caregiver (family members) on the relationship between health capacity and health function (*B* = 1.19, S. E. = 0.50, *p* = 0.016).

**Table 2:** The moderator examinations of environmental factors and individual characteristics in the relationship between heath capacity and health function.

Variables	*B*	S. E.	Beta	*t*	*p*	95% CI of *B*	
						Lower	Upper
The moderator examination model of environmental factors							
Health capacity	0.796	0.454	0.063	1.754	0.08	−0.094	1.685
Age-friendly social environment	3.287	0.229	0.257	14.325	<0.001	2.837	3.737
APGAR	1.661	0.211	0.126	7.881	<0.001	1.247	2.074
Daily affairs support from family members	−0.928	0.465	−0.03	−1.995	0.046	−1.84	−0.016
Economic support from family members	−0.838	0.461	−0.028	−1.816	0.069	−1.742	0.067
Emotional support from family members	−0.755	0.37	−0.03	−2.038	0.042	−1.482	−0.029
Personal expectations of later life	0.931	0.189	0.074	4.932	<0.001	0.561	1.301
Health capacity * Age-friendly social environment	−0.852	0.181	−0.078	−4.705	<0.001	−1.207	−0.497
Health capacity * APGAR	0.726	0.199	0.062	3.651	<0.001	0.336	1.116
Health capacity * Daily affairs support from family members	0.137	0.372	0.008	0.369	0.712	−0.592	0.866
Health capacity * Economic support from family members	0.294	0.163	0.028	1.802	0.072	−0.026	0.614
Health capacity * Emotional support from family members	0.346	0.455	0.024	0.76	0.447	−0.546	1.238
Health capacity * Personal expectations in later life	−0.003	0.452	0	−0.006	0.995	−0.888	0.883

Each model of linear regressions was conducted after controlling for variables, and the model residuals and collinearity tests met the standards. Due to space limitations, the results of constant terms, covariates, residuals, and collinearity tests are not presented in the article. All of continuous variables were centralizing in the models.

### Pathway analysis of how health capacity and environmental factors work together on health function

After the explorations of determinants of health function, a SEM model was constructed with appropriate goodness-of-fit (see Supplementary Table 4 in Part C in the Appendix). Figure 1, Table 3 and Supplementary Table 5 in the Appendix show the results of path analysis. The total effect of health capacity on health function is path d = 0.348 (*p*<0.001), and the direct effect is path d’ = 0.079 (*p*<0.001). The indirect effect paths comprising of 3 significant ways (*p*<0.001) are as follows: path a1 (health capacity→APGAR) = 0.337 and path a2 (APGAR→health function) = 0.141, path b1 (health capacity→age-friendly social environment) = 0.517 and path b2 (age-friendly social environment→health function) = 0.409, and path c1 (health capacity→personal expectation in later life) = 0.216 and path c2 (personal expectation in later life→health function) = 0.047. The indirect pathway’s coefficient of health capacity is 0.269 higher than the direct pathway’s. The interaction terms of health capacity with APGAR, age-friendly social environment, and main caregiver are all significant in this model (*p*<0.01). Among these, the interaction term between health capacity and age-friendly social environment has a significant negative effect on health function (*β* = −0.059, *p* = 0.002). Taking older adults into two groups (higher scores in social environment vs. lower scores in social environment), the study found that the negative moderating role of age-friendly environment appears in older adults reporting lower scores in social environment (see Part C in the Appendix).

**Table 3:** The pathway effects of health capacity and environmental factors on health function.

Independent variables	Pathway	Dependent variables	Coefficient	*p*
Health capacity	→	APGAR	0.337	<0.001
Health capacity	→	Age-friendly social environment	0.517	<0.001
Health capacity	→	Personal expectations in later life	0.216	<0.001
Health capacity	→	Health function	0.079	<0.001
APGAR	→	Health function	0.141	<0.001
Age-friendly social environment	→	Health function	0.409	<0.001
Personal expectations in later life	→	Health function	0.047	0.001
Health capacity * APGAR	→	Health function	0.062	<0.001
Health capacity * Age-friendly social environment	→	Health function	−0.059	0.002
Health capacity * Main caregiver	→	Health function	0.074	<0.001
Main caregiver	→	Health function	−0.272	<0.001

The model is estimated using Maximum Likelihood Estimation (ML), which is the most commonly used parameter method in path analysis due to its independence from measurement units. The overall adaptation of the model is good, and the adaptation standards of the main fitting indicators have been met.

## Discussion

The purpose of this study was to explore the determinants of older adults’ health function from a full perspective of health capacity and environmental factors and specifically examine how health capacity and environmental factors work together on health function. Our findings highlight three key points. First, both individual health capacity and family and social environments were drivers of health function. Second, environmental factors acted as mediators and moderators in the relationship between health capacity and health function. Among these, age-friendly family (APGAR) and social environments, and personal expectations in later life partially mediated the correlation between health capacity and health function. Third, in the pathway to health function, the age-friendly social environment played a predominant role, followed by health capacity, APGAR, and personal expectations in later life.

### Multi-layer determinants of health function

Align with previous findings (26–28), the present results suggest that both an individual’s inherent characteristics and surrounding characteristics have associations with health function in aging settings. A higher level of health capacity was associated with a higher level of health function. Specifically, cognitive ability and health choice ability were positively associated with all of the health function. Reasons about specific capacity associations may as follow. Cognitive ability is acknowledged as one of the most fundamental factors in later life. It provides a basic ability for older adults to keep being correctly aware of the time, space, and interpersonal relationships, which further draws upon the linkage between older adults and their living surroundings (29). Cognitive ability gives older adults a confirmation of their existence. And the key role of cognitive ability in maintaining older adults’ health has been largely studied. It was found to be able to predict 5-year mortality and 10-year mortality (30). A systematic review found that older adults with cognitive impairment were more likely to get functional disability (31). These supported the finding in this study. Health choice ability refers to one’s proactive action in the life course. Older adults with higher health choice ability tend to be more active in health and participation. Existing findings indicated that active participation had a positive relationship with health-related quality of life (32). Thus, it can be inferred that cognitive ability and health choice ability may contribute to the sense of control of one’s life, leading to the motivations to valued beings/states. Besides health capacities, multi-layer environmental factors had positive associations with showed potential in promoting health function. Consistent with previous studies, older adults could benefit from family and social support in multiple health outcomes (33). In addition, our results found that a higher level of life expectation was positively associated with better health function, which supported Active Aging initiatives.

Besides APGAR, the three specific family assistance were not associated with health function. One possible explanation is that family assistance represents the one-way reception. As reciprocity theory says, receiving when incapable of giving may raise doubt about an individual’s usefulness in a relationship (34–36), which may trigger psychological discomfort. This calls for attention to the interaction with older adults when creating an age-friendly environment, especially in the family context.

### Pathway of health capacity and environmental factors to health function

This study firstly explored the pathway of how health capacity and multi-layer environments work together on health function. Our findings suggest that health capacity worked mainly through environmental factors on health function, and age-friendly social environment played the biggest role. There are several lines of explanation for these pathways. Family is the proximal environment that people directly contact with. Older adults who reported higher scores in APGAR were more likely to experience tight family cohesion and respect, which may enhance one’s desire to use their capacity and then facilitate one’s function. And among all the demographic characteristics, only main caregiver demonstrated a positive moderating role in the relationship between health capacity and health function. This result also reflects the strengthening role of interactive family functions. Society is the distal environment that older adults live in. An age-friendly social environment is defined as the accessibility and affordability of infrastructure, as well as the participation and belonging in society. Previous studies found that a good walking environment could enhance one’s mobility (37), thereby preventing disability. The age-friendly social environment could be seen as an empowerment tool for older adults to utilize capacities. This study pointed the facilitator and empowerment role of multi-layer environments in the relationship between health capacity and health function. Our study also extended prior evidence by distinguishing the role of family and society in the aging health context. There has been many arguments on the role of family. Family conflict and harmony are always intertwined, reflecting the complexity of family function (38, 39). To some extent, the tight support from nearby may evoke a sense of being controlled. On the contrary, current evidence of the role of social environment showed a consistent trend. Age-friendly social environment has been proven to be positive for older adults’ physical health and mental health (40). Some authors have speculated that an age-friendly social environment could wipen opportunities in old age by improving mobility, ensuring participation, guaranteeing belongings, and improving living standards, thereby preventing functional decline (41). These may explain the different role of family and social environment in the association between health capacity and health function.

Furthermore, the study found that the age-friendly social environment was negatively associated with health function among older adults with lower health capacity. This result was to some extent consistent with social exclusion opinions. Some researchers found out an unexpected negative psychological burden in the health limited group. Older adults with poor health were more likely to experience social exclusion (42). The feelings of exclusion is subtle and may come from the personal identification. For example, fraily identify could prevent people from participating in an inclusive community (43). Typically in Asian context, “mianzi,” which refers to one’s dignity, was the driving force behind many behaviors. Using the age-friendly tools sometimes was seen as a behavior of losing “mianzi.” And another explanation of this result maybe the universal design of age-friendly social environment. The friendliness of its design is not sufficient to meet the special needs of people with functional impairments (44).

Some limitations of this study should be addressed. First, these findings are limited by the use of a cross-sectional analysis and the sample from Zhejiang Province. The generalizability and applicability of this result need to be noted. Based on our evidence, future work, for instance, in the context of longitudinal analysis or intervention studies, is needed to draw a full insight into the drivers and pathways to healthy aging. Second, a self-report assessment of the environmental variables, which may be affected by self-desirability, was adopted in this study. Although the efficacy of how people perceive the age-friendliness of surroundings has been examined (45), future work using multiple-methods assessment (including objective construction and subjective perception) is encouraged. Third, we used a composite factor of health capacity and the age-friendly social environment in the pathway to health function. This may limit the complete focus on these determinants. As the first study that observed the multi-layer determinants and their effects on health function, the composite factor could provide a vivid glimpse of pathways to healthy aging. We call for further efforts on the effect chain of some specific factors related to healthy aging.

## Conclusion

Our findings contribute to the sparse literature that multi-layer determinants (capacity and environment) could work together on health function. We posit that health capacity had both direct and indirect associations with health function. And the indirect associations through age-friendly environments has proven to be predominant. Among environmental factors, an age-friendly social environment takes up a key role in the pathway to health function. Further efforts on the effect chain of specific capacity and environmental factors are needed.

## Data Availability

The raw data supporting the conclusions of this article will not be made available, until the whole work about the healthy ageing research has been completed.
