# Supplementary material for: Environmental and capacity drivers of health function: a full perspective of the healthy aging framework
Source: Front Public Health. 2025 Sep 24;13:1659061. doi: 10.3389/fpubh.2025.1659061 (PMC12504343; doi:10.3389/fpubh.2025.1659061)
Supplement: Supplementary file 1 [file Table_1.DOCX]

**Appendix**

**Part A** Measures to assure quality data

To ensure data quality, several measures were implemented: 1) Investigator Training. Prior to the formal survey, all investigators underwent three intensive training sessions. These included a detailed explanation of the study purpose, item-by-item review of the questionnaire, and situational simulation exercises. Furthermore, during the pilot phase, we held debriefing sessions every half-day to refine the survey protocol and update the training manual iteratively until no new issues emerged. As a result, by the time of the formal survey, all investigators had become highly familiar with the questionnaire and mastered appropriate communication techniques, thereby substantially ensuring data completeness. 2) Local Collaboration. To enhance trust and participation among elderly respondents, all interviews were conducted with the accompaniment of frontline staff from local health or civil affairs departments. Their presence helped establish rapport and encouraged more open and accurate responses.

**Part B** The cross-scale validation of the comprehensive assessment scale for health and ageing care needs

After reviewing a total of 33 scales that measure the health states of older adults (e.g. OARS-MFAQ, FAI, LLFDI, HAQ, WHOQOL-OLD), we found these scales put focus on the overall health states of older adults while not distinguishing health capacity from health function. So we chose the Comprehensive Assessment Scale for Health and Ageing Care Needs as study tool.

In order to validate this scale, we adopted WHOQOL-OLD and EQ-5D-3L as the validated scales for this study tool. Considering there is a lack of cognition part in the above two scales, we selected Mini-cog as the validated scale for the cognition part in the study tool. STable 1 shows the results of validation.

**STable 1 The validation results of cross-scale analysis**

|  | Physical health function | Overall perception of life health | Mental health function | Psycho-  emotional function | Cognitive ability | Care-seeking ability | Health choice ability | The total scale |
| --- | --- | --- | --- | --- | --- | --- | --- | --- |
| WHOQOL-OLD | 0.40** | 0.28** | 0.35** | 0.37** | 0.17** | -0.06* | 0.23** | 0.47** |
| EQ-5D-3L | 0.68** | 0.36** | 0.45** | 0.35** | 0.22** | -0.01 | 0.41** | 0.66** |
| mini-cog | 0.29** | 0.14** | 0.22** | 0.21** | 0.74** | 0.09** | 0.40** | 0.44** |

Note. *=p＜0.05，**=p＜0.01.

The total scale is moderately correlated with WHOQOL-OLD and strongly correlated with EQ-5D-3L. Most of the health function dimensions of the total scale, such as physical health function, overall perception of life health, mental health function, and psycho-emotional function, are moderately correlated with the two calibration scales (p<0.01). Cognitive ability, care-seeking ability, and health choice ability are not included in the conceptual scope of WHOQOL-OLD and EQ-5D-3L. The Spearman correlation coefficient between cognitive ability dimension and mini-cog scale is 0.74, significantly higher than the correlation coefficients between other dimensions and mini-cog scale, showing a strong correlation. Overall, the criterion validity of the self-designed scale is acceptable. Thus, we finally used the Comprehensive Assessment Scale for Health and Ageing Care Needs in this study. And the further validation and adoption of this scale is needed.

**Part C** The descriptive analysis of health function and health capacity

**STable 2 The descriptive results of health function and health capacity across demographic characteristics (n=2688)**

| **Variables** | **Health Function** | | | **Health Capacity** | | |
| --- | --- | --- | --- | --- | --- | --- |
|  | Median（IQR） | χ²/Z | *P* | Median（IQR） | χ²/Z | *P* |
| **Socio-demographic characteristics** |  |  |  |  |  |  |
| Age |  | 237.25 | ＜0.001 |  | 110.11 | ＜0.001 |
| 60-69 | 62（13） |  |  | 27（8） |  |  |
| 70-79 | 60（14） |  |  | 27（7） |  |  |
| ≥80 | 50（21） |  |  | 24（9） |  |  |
| Gender |  | -3.43 | 0.001 |  | -0.61 | 0.545 |
| Male | 61（16） |  |  | 26（8） |  |  |
| Female | 59（16） |  |  | 27（8） |  |  |
| Ethics |  | -0.63 | 0.529 |  | -1.39 | 0.165 |
| Han | 60（17） |  |  | 26（8） |  |  |
| The others | 60（12） |  |  | 25（7） |  |  |
| Residential areas |  | -7.74 | ＜0.001 |  | -21.29 | ＜0.001 |
| Rural | 58（19） |  |  | 24（8） |  |  |
| Urban | 61（14） |  |  | 29（6） |  |  |
| Socioeconomic status |  | 172.22 | ＜0.001 |  | 551.85 | ＜0.001 |
| Low | 56（20） |  |  | 23（8） |  |  |
| Moderate | 60（15） |  |  | 26（7） |  |  |
| High | 63（11） |  |  | 29（5） |  |  |
| Living arrangement |  | -6.19 | ＜0.001 |  | -3.57 | ＜0.001 |
| With Household Member(s) | 60（16） |  |  | 27（8） |  |  |
| Alone | 56（16） |  |  | 25（7） |  |  |
| Marital status |  | -12.44 | ＜0.001 |  | -10.51 | ＜0.001 |
| Married | 61（14） |  |  | 27（7） |  |  |
| Widowed or divorced or never married | 54（19） |  |  | 24（8） |  |  |
| **Aged care characteristics** |  |  |  |  |  |  |
| Main caregivers |  | 508.33 | ＜0.001 |  | 236.97 | ＜0.001 |
| By self | 61（12） |  |  | 27（8） |  |  |
| Family members | 41（20） |  |  | 22（9） |  |  |
| Others | 36（15） |  |  | 21（7） |  |  |
| Enough source of livelihood |  | 361.89 | ＜0.001 |  | 377.52 | ＜0.001 |
| No | 46（27） |  |  | 23（9） |  |  |
| Even | 59（16） |  |  | 25（8） |  |  |
| Yes | 63（11） |  |  | 29（6） |  |  |
| Aged care type |  | -9.53 | ＜0.001 |  | -6.4 | ＜0.001 |
| Ageing in place | 60（16） |  |  | 27（8） |  |  |
| Ageing in institutions | 38（14） |  |  | 21（8） |  |  |
| **Health related characteristics** |  |  |  |  |  |  |
| Chronic diseases |  | 360.74 | ＜0.001 |  | 8.4 | 0.015 |
| None | 64（9） |  |  | 26（8） |  |  |
| 1 | 62（13） |  |  | 27（7） |  |  |
| ≥2 | 54（20） |  |  | 26（8） |  |  |
| Doctor visit in the past 2 weeks |  | -7.82 | ＜0.001 |  | -0.1 | 0.92 |
| No | 60（16） |  |  | 27（8） |  |  |
| Yes | 54（18） |  |  | 27（8） |  |  |
| Hospitalization in the past 1 year |  | -14.1 | ＜0.001 |  | -7.37 | ＜0.001 |
| No | 61（14） |  |  | 27（7） |  |  |
| Yes | 50（22） |  |  | 25（9） |  |  |
| Fall in the past 1 year |  | 275.32 | ＜0.001 |  | 160.57 | ＜0.001 |
| None | 61（13） |  |  | 27（7） |  |  |
| 1 | 55（21） |  |  | 25（9） |  |  |
| ≥2 | 38（21） |  |  | 21（6） |  |  |

Note. IQR=75% percentile -25% percentile. Considering the abnormal distribution of the data, the Mann Whitney U test was used for the distribution of health function or health capacity in binary variables, and the test statistic was the Z-value; The Kruskal-Wallis test is used for the distribution of health function or health capacity in the three categorical variables, with the test statistic being the χ² value. Socioeconomic status was analyzed using principal component analysis after centralizing three variables: education achievement, occupation before retirement, and monthly disposable income, and the resulting composite index was divided into low, medium, and high levels according to the third percentile.

STable 3 presents the bivariate correlations between health capacity, environmental factors, and health function. Health capacity (ρ=0.28, p＜0.001), personal expectations of later life (Z=0.28, p＜0.001), APGAR (ρ=0.31, p＜0.001), and the age-friendly social environment (ρ=0.54, p＜0.001) are positively related to health function. Economic support from family members is negatively associated with health function (Z=-6.92, p＜0.001).

**STable 3 Bivariate correlation results between health capacity factors, environmental factors, and health function**

| **Variables** | Health function (total score) | Physical health function | Mental health function | Psycho-emotional function | Overall perception of life health |
| --- | --- | --- | --- | --- | --- |
| **Health capacity factors** | | | | | |
| Health capacity （total score) | 0.28 | 0.42 | 0.30 | 0.22 | 0.22 |
| *P* | ＜0.001 | ＜0.001 | ＜0.001 | ＜0.001 | ＜0.001 |
| Cognitive ability | 0.28 | 0.27 | 0.19 | 0.19 | 0.14 |
| *P* | ＜0.001 | ＜0.001 | ＜0.001 | ＜0.001 | ＜0.001 |
| Care-seeking ability | 0.01 | 0.00 | 0.07 | -0.06 | 0.01 |
| *P* | 0.813 | 0.866 | ＜0.001 | 0.002 | 0.618 |
| Health choice ability | 0.41 | 0.42 | 0.28 | 0.23 | 0.22 |
| *P* | ＜0.001 | ＜0.001 | ＜0.001 | ＜0.001 | ＜0.001 |
| **Environmental factors** | | | | | |
| Personal expectations in later life | 0.24 | 0.21 | 0.21 | 0.14 | 0.21 |
| *P* | ＜0.001 | ＜0.001 | ＜0.001 | ＜0.001 | ＜0.001 |
| APGAR | 0.31 | 0.24 | 0.34 | 0.24 | 0.20 |
| *P* | ＜0.001 | ＜0.001 | ＜0.001 | ＜0.001 | ＜0.001 |
| Daily affairs support from family members | -1.55 | -2.27 | -2.32 | -3.23 | -0.11 |
| *P* | 0.122 | 0.023 | 0.020 | 0.001 | 0.909 |
| Economic support from family members | -6.92 | -7.64 | -2.05 | -5.83 | -3.22 |
| *P* | ＜0.001 | ＜0.001 | 0.040 | ＜0.001 | 0.001 |
| Emotional support from family members | -0.41 | -0.39 | -2.27 | -4.36 | -1.66 |
| *P* | 0.684 | 0.697 | 0.023 | ＜0.001 | 0.097 |
| Age-friendly social environment | 0.54 | 0.54 | 0.37 | 0.27 | 0.33 |
| *P* | ＜0.001 | ＜0.001 | ＜0.001 | ＜0.001 | ＜0.001 |

Note. Considering the abnormal distribution of the data, the Spearman analysis was used to examine the relationship between health function and continuous variables, and the test statistic was the ρ-value. The Mann Whitney U test was used to explore the relationship between health function and categorical variables, with the test statistic being the Z-value.

**Part D** The indices and results of pathway analysis of how health capacity and environmental factors work together on health function

The model is estimated using Maximum Likelihood Estimation (ML), which is the most commonly used parameter method in path analysis due to its independence from measurement units. The overall adaptation of the model is good, and the adaptation standards of the main fitting indicators have been met, as shown in STable 4.

**STable 4 The goodness-of-fit of the pathway analysis model**

| **Main indices** | **The standard criteria** | **Index** | **Fit or Not** |
| --- | --- | --- | --- |
| Absolute fit indices |  |  |  |
| SRMR | ＜0.05 | 0.022 | Fit |
| RMSEA | ＜0.08 | 0.039 | Fit |
| GFI | ＞0.90 | 0.997 | Fit |
| AGFI | ＞0.90 | 0.981 | Fit |
| CNIN/DF | <3.00 is excellent,<5.00 is good,<8.00 is acceptable | 4.453 | Fit |
| Relative fit indices |  |  |  |
| TLI | ＞0.90 | 0.980 | Fit |
| CFI | ＞0.90 | 0.997 | Fit |

STable 5 shows the overall pathway effects of each determinants to health function

**STable 5 The overall pathway effects of each determinants**

|  | Direct effect | Indirect effect | Total effect |
| --- | --- | --- | --- |
| Health capacity | 0.079 | 0.269 | 0.348 |
| APGAR | 0.141 | 0 | 0.141 |
| Age-friendly social environment | 0.409 | 0 | 0.409 |
| Personal expectations in later life | 0.047 | 0 | 0.047 |
| Health capacity * APGAR | 0.062 | 0 | 0.062 |
| Health capacity * Age-friendly social environment | -0.059 | 0 | -0.059 |
| Health capacity * Main caregiver | 0.074 | 0 | 0.074 |
| Main caregiver | -0.272 | 0 | -0.272 |

STable 6 shows the role of age-friendly social environment in different level of health capacity

**STable 6 The moderation model of social environment and health capacity**

| Model/Variables | B | S.E. | p |
| --- | --- | --- | --- |
| Model 1（Lower health capacity group） |  |  |  |
| Health capacity * Social environment | -0.796 | 0.318 | 0.012 |
| Model 2（Higher health capacity group） |  |  |  |
| Health capacity * Social environment | -0.666 | 0.628 | 0.289 |

Note. The dependent variable is the total score of health function. Both models included variables that have been validated to have mediating and moderating effects, and controlled for general demographic information characteristics. Due to space limitations, they are not presented in the article. All continuous variables and product terms have been centralized.

STable 7 showes the moderating role of demographic factors in the relationship between health capacity and health function. We carefully considered testing the moderating role of health risk factors (e.g., chronic conditions). However, upon investigation, we identified a high degree of multicollinearity between these risk factors and the core constructs of our model (health capacity and health function). Introducing interaction terms under these conditions would not only yield unstable and unreliable parameter estimates but also complicate the interpretation of the main effects. Therefore, to maintain the statistical integrity and parsimony of our model, we deemed it methodologically unsound to include these moderation effects.

**STable 7** The moderating role of demographic factors in the relationship between health capacity and health function

| Variable | B | S.E. | Beta | t | p | 95%CI of B | |
| --- | --- | --- | --- | --- | --- | --- | --- |
|  |  |  |  |  |  | Lower | Upper |
| Health capacity*Gender | 0.547 | 0.360 | 0.075 | 1.521 | 0.129 | -0.158 | 1.252 |
| Health capacity*Age (70-79) | -0.203 | 0.407 | -0.010 | -0.499 | 0.618 | -1.001 | 0.595 |
| Health capacity*Age (≥80) | 0.492 | 0.469 | 0.021 | 1.051 | 0.293 | -0.427 | 1.411 |
| Health capacity*Register | 0.251 | 0.399 | 0.030 | 0.628 | 0.530 | -0.532 | 1.034 |
| Health capacity*SES (Moderate) | 0.374 | 0.440 | 0.016 | 0.850 | 0.395 | -0.489 | 1.237 |
| Health capacity*SES (High) | 0.216 | 0.534 | 0.009 | 0.404 | 0.686 | -0.831 | 1.263 |
| Health capacity*Marital status | 0.502 | 0.388 | 0.056 | 1.293 | 0.196 | -0.259 | 1.263 |
| Health capacity*Living arrangement | 0.440 | 0.478 | 0.013 | 0.920 | 0.358 | -0.497 | 1.376 |
| Health capacity*Maincaregiver(by family) | 1.191 | 0.496 | 0.045 | 2.403 | 0.016 | 0.219 | 2.163 |
| Health capacity*Maincaregiver(by others) | 1.654 | 1.107 | 0.026 | 1.494 | 0.135 | -0.518 | 3.826 |
| Health capacity*livelihood (even) | -0.219 | 0.497 | -0.011 | -0.441 | 0.659 | -1.193 | 0.755 |
| Health capacity*livelihood (enough) | -0.514 | 0.573 | -0.023 | -0.897 | 0.370 | -1.636 | 0.609 |
| Health capacity*Aged care type | -0.097 | 1.044 | -0.008 | -0.093 | 0.926 | -2.145 | 1.95 |

Note. Each model incorporates transformation factors that have been validated to have mediating and moderating effects, while controlling for other demographic information characteristics; The model residuals and collinearity tests meet the standards. Due to space limitations, the results of constant terms, covariates, residuals, and collinearity tests are not presented in the article.
